# Estimated dietary phytoestrogen intake and major food sources among women during the year before pregnancy

**DOI:** 10.1186/1475-2891-10-105

**Published:** 2011-10-06

**Authors:** Suzan L Carmichael, Amparo G Gonzalez-Feliciano, Chen Ma, Gary M Shaw, Mary E Cogswell

**Affiliations:** 1 Department of Pediatrics, Stanford University, Stanford, CA, USA; 2 Division of Birth Defects and Developmental Disabilities, currently with Science Applications International Corporation, Immunization Services Division, Centers for Disease Control and Prevention, Atlanta, GA, USA; 3 Division of Birth Defects and Developmental Disabilities, currently with Heart Disease and Stroke Prevention, Centers for Disease Control and Prevention, Atlanta, GA, USA

**Keywords:** phytoestrogen, lignan, isoflavone, pregnancy

## Abstract

**Background:**

Phytoestrogens may be associated with a variety of different health outcomes, including outcomes related to reproductive health. Recently published data on phytoestrogen content of a wide range of foods provide an opportunity to improve estimation of dietary phytoestrogen intake.

**Methods:**

Using the recently published data, we estimated intake among a representative sample of 6,584 women of reproductive age from a multi-site, population-based case-control study, the National Birth Defects Prevention Study (NBDPS). The NBDPS uses a shortened version of the Willett food frequency questionnaire to estimate dietary intake during the year before pregnancy. We estimated intake among NBDPS control mothers.

**Results:**

Lignans contributed 65% of total phytoestrogen intake; isoflavones, 29%; and coumestrol, 5%. Top contributors to total phytoestrogen intake were vegetables (31%) and fruit (29%); for isoflavones, dairy (33%) and fruit (21%); for lignans, vegetables (40%) and fruit (29%); and for coumestans, fruit (55%) and dairy (18%). Hispanic women had higher phytoestrogen intake than non-Hispanic white or black women. Associations with maternal age and folic acid-containing supplements were more modest but indicated that older mothers and mothers taking supplements had higher intake.

**Conclusions:**

The advantage of the approach used for the current analysis lies in its utilization of phytoestrogen values derived from a single laboratory that used state-of-the-art measurement techniques. The database we developed can be applied directly to other studies using food frequency questionnaires, especially the Willett questionnaire. The database, combined with consistent dietary intake assessment, provides an opportunity to improve our ability to understand potential associations of phytoestrogen intake with health outcomes.

## Introduction

Phytoestrogens are plant substances that are structurally and functionally comparable to 17-beta estradiol and capable of producing estrogenic effects [1]. They are particularly high in certain foods, such as soy products and certain nuts and seeds. They may also have anti-estrogenic and anti-androgenic effects [2-5]. As such, phytoestrogens have been likened to natural selective estrogen receptor modulators [4]. They may protect against a number of health outcomes, including certain cancers, diabetes, and cardiovascular disease [2,6-10]. In addition to their endocrine-related activities, their anti-proliferative and anti-oxidant properties may also contribute to their associations with health outcomes [6,11]. Several recent animal studies indicate either teratogenic or protective effects of phytoestrogens with respect to fetal development [12-16].

Estimating dietary intake of phytoestrogens has been a challenge for previous studies. Phytoestrogen values have been unavailable for many foods, and measurement techniques have had inherent limitations. Recent improvements in analytic techniques [17] and extensive data recently published by Kuhnle et al. [18-21] provide an opportunity to improve estimation of dietary phytoestrogen intake. Using these recently published data, we estimated intake among a representative sample of women of reproductive age from a multi-site, population-based case-control study, the National Birth Defects Prevention Study (NBDPS) [22]. The NBDPS includes mothers of cases with over 30 different types of birth defects and mothers of control infants who did not have any major structural malformations. The NBDPS uses a shortened version of the well-known Willett food frequency questionnaire to estimate dietary intake during the year before pregnancy.

Our objective was to estimate intake of the phytoestrogens - lignans, isoflavones, and coumestans - and specific compounds within these groups (e.g., genistein), among control mothers participating in the NBDPS. In this paper we describe our approach to assigning phytoestrogen values to the food items queried in the NBDPS. Additionally, we describe the main contributors to phytoestrogen intake and maternal characteristics associated with intake.

## Methods

### Study design

The NBDPS is a multi-state case-control study of more than 30 different birth defects. It began with deliveries that had estimated due dates in October, 1997. Data collection is on-going. The study is an approved activity of the Institutional Review Boards of the participating study centers and the Centers for Disease Control and Prevention. Detailed study methods and descriptions of the surveillance systems in the ten states that contributed data to this analysis have been published [23,24]. The current analysis is restricted to mothers of controls. Each state randomly selected approximately 100 non-malformed, live born controls per study year from birth certificates (AR 2000-2005, GA 2001-2005, IA, MA, NC, NJ, UT) or from birth hospitals (AR 1997-1999, CA, GA 1997-2000, NY, TX) to represent the population from which the cases were derived. Maternal interviews were conducted using a standardized, computer-based questionnaire, primarily by telephone, in English or Spanish, no earlier than six weeks after the infant's estimated date of delivery and no later than 24 months after the estimated due date. Exposures to a variety of factors were assessed, relative to the woman's estimated date of conception, which was derived by subtracting 266 days from the woman's expected due date. The expected due date was based on mother's self-report; if unknown, it was estimated from information in the medical record (less than two percent of subjects).

### Food frequency questionnaire

Interviewers asked women about their average intake of foods using a shortened version of the food frequency questionnaire (FFQ) developed by Willett and colleagues that included 58 food items [25]. Participants reported how often, on average, they had consumed the food items in the year before they became pregnant. For seasonal foods, such as fruits and vegetables, they averaged their intake over the six months prior to pregnancy. Fifteen response categories were possible ranging from once per month to 6 or more per day. Foods they ate less than once a month were recorded as "never or none."

Intake of breakfast cereals and food supplements (e.g., protein powders added to beverages) were assessed by separate, more detailed questions, which covered intake during the three months before pregnancy through delivery (assessed by month, except by trimester for the second and third trimesters of pregnancy). For each cereal and food supplement they consumed, they reported the brand name, time periods when they consumed it (by month or trimester), and average frequency of intake. Few women consumed food supplements (n = 674), and phytoestrogen values were not available for them, so they were not included in the phytoestrogen calculations. Women answered detailed questions about their intake of sodas during the year before pregnancy; we assumed sodas did not contain phytoestrogens. The USDA version 19 national nutrient database was the source of non-phytoestrogen nutrient values [26]. Dietary folate intake was expressed as dietary folate equivalents (DFEs), which was derived by multiplying the amount of folic acid from fortified foods by 1.7 to account for its greater bioavailability and then adding that amount to natural folate from foods.

Women answered detailed questions about their frequency of intake of *caffeinated *coffee and tea during the year before pregnancy. Because the information on intake of coffee and tea does not include decaffeinated coffee and tea, it is not included the main analyses, but descriptive information on phytoestrogen intake among women who drank caffeinated coffee or tea is provided.

### Phytoestrogen database construction

To create the database, we used phytoestrogen values published by the Kuhnle laboratory [18-21] given the advantages that the values were recent, derived from a single laboratory using state-of-the-art techniques [17], available for a relatively consistent set of specific phytoestrogens across all food items, and available for most of the foods in our FFQ. The Kuhnle laboratory processed multiple samples for each food item that was analyzed, selecting samples that represented different manufacturers, varieties, and countries of origin to the extent possible from local food outlets. Detailed information on the database we developed is available upon request.

To create the database, three authors (SC, AG, MC) reviewed each food item from the FFQ individually. For 25 food items, phytoestrogen values were derived from equivalent foods (e.g., milk, broccoli). For 14 items, we assigned the most frequently eaten food, using data from the National Health and Nutrition Examination Survey as reference [27]; e.g., values for tomatoes were used for the food item "tomatoes or tomato juice". For eight food items, we calculated values based on combinations of foods: a combination of fat and lean beef and pork for three meat-related food items; a combination of noodles and rice for "rice or pasta," since their frequency of consumption is relatively similar at the population level [27]; and simple recipes for pie and salsa. For nine food items, we used proxy foods because phytoestrogen values for equivalent foods were not available; e.g., pig liver for organ meats, and flour for breads and tortillas. Regular and whole wheat flour were used as proxies for breads and tortillas because the Kuhnle et al. values were likely elevated due to the addition of soy flour to breads that were included in their databases [21]. We assigned a value of zero to two food items, "chocolate" and "candy without chocolate," under the assumption that these items contain zero to negligible phytoestrogens.

For cereals, we assigned the closest match available from the Kuhnle publication on cereals [21]. If the cereal name reported in NBDPS was not an obvious match to a cereal reported by Kuhnle et al., we selected the cereal that was the best match, giving priority to grain content, then fiber content, then typical serving size. We found information on these characteristics through web-based searches for product labels and manufacturer information. For more difficult matches, we prioritized the key words in the cereal name as reported in NBDPS in the order that they appeared in the mother's response. For responses that did not provide helpful key words for matching, we assigned values that were an average of Kuhnle's values for oat bran flakes and corn flakes, given that in the NBDPS, the most commonly reported cereals were Cheerios (26.9%) and corn flakes or frosted flakes (28.2%). Oat bran flakes rather than multigrain hoops was considered the closest match for Cheerios because in the U.S. Cheerios tend to be oat-based.

Kuhnle et al.'s articles on drinks and nuts; fruits and vegetables; and cereals reported on a consistent set of five isoflavones, two lignans, and the coumestan, coumestrol [18,20,21]. The articles on drinks and nuts and fruits and vegetables included totals for phytoestrogens, isoflavones and lignans. We calculated the totals from the article on cereals. The article on animal products reported total isoflavones, total lignans, and coumestrol, plus values for other phytoestrogens equol, enterolactone, and enterodiol [19]. Therefore, for animal products we used from this article, we calculated total phytoestrogens ourselves as a sum of isoflavones, lignans, and coumestrol, since no other article had information on the other reported phytoestrogens.

### Analyses

We restricted analyses of phytoestrogen intake to the 6,789 mothers of control infants who had estimated due dates from October 1997 to December 2005. After excluding 90 mothers whose energy intake was < 500 or > 5000 kilocalories, 46 mothers who had more than one missing food item, and 69 mothers who fit both criteria (these extremes may reflect invalid data), 6,584 mothers were available for analysis.

We examined the distribution of intake of phytoestrogens and which food groups and food items contributed the most to their consumption, overall and by race-ethnicity. We also examined whether 'high' or 'low' total phytoestrogen intake (defined as intake in the highest or lowest quartile) was associated with selected maternal characteristics. Specifically, we ran two multivariable logistic regression models to estimate odds ratios and 95 percent confidence intervals associated with high or low intake, relative to intake in the middle two quartiles. The models included maternal race-ethnicity and nativity (non-Hispanic white, Hispanic, African-American, and other (which includes mixed races), education (less than, equal to or greater than high school education), age (< 25, 25-34, or 35 or more years old), intake of folic acid-containing supplements (intake began before pregnancy, during the first trimester of pregnancy, or later or not at all from three months before and during pregnancy), and energy intake (kcals). Maternal characteristics were self-reported during the interviews. Folic acid-containing supplements largely consisted of prenatal multivitamin/mineral formulations [28].

## Results

The majority of the control mothers were non-Hispanic white (61%), had greater than a high school education (59%), were 25-34 years old (53%), and began taking folic acid-containing supplements during the three months before or first three months of pregnancy (88%).

Information on the distribution of estimated phytoestrogen intake is provided in Table 1 for the 6,584 women in our study group. Overall, lignans contributed 65% of total phytoestrogen intake; isoflavones, 29%; and coumestrol, 5%. Among the measured lignans, secoisolariciresinol accounted for 83% of intake and matairesinol for 18%. Among the isoflavones, genistein accounted for 35%, biochanin A for 23%, daidzein for 19%, glycetin for 14% and formononetin for 9% of intake. Correlations of the phytoestrogen sub-types with each other ranged from 0.6-0.8 (Table 2). Correlations with energy intake, carbohydrates, and fiber were also high, 0.6-0.9, whereas correlations with folate were lower, approximately 0.5.

**Table 1:** Distribution of dietary intake of phytoestrogens (μg/day) among 6,584 mothers of control infants in the National Birth Defects Prevention Study.*

Phytoestrogen	Mean ± SD	Median (Interquartile range)
Total Isoflavones	58.5 ± 35.0	49.1 (35.2, 72.2)
Genistein	23.7 ± 13.7	20.3 (14.4, 29.5)
Biochanin A	15.2 ± 9.1	13.1 (9.1, 18.8)
Daidzein	12.9 ± 8.9	10.4 (7.0, 16.0)
Glycetin	9.7 ± 7.5	7.5 (5.1, 11.8)
Formononetin	5.7 ± 3.2	5.0 (3.5, 7.1)
Total Lignans	139.0 ± 98.2	114.2 (75.6, 173.4)
Secoisolariciresinol	117.3 ± 87.2	94.8 (61.1, 146.9)
Matairesinol	22.4 ± 16.9	18.0 (12.4, 27.0)
Coumestrol	10.0 ± 6.6	8.5 (5.4, 12.7)
Total Phytoestrogens	208.9 ± 133.6	174.8 (121.2, 256.5)

* Does not include intake from coffee or tea or alcoholic beverages.

**Table 2:** Correlations among dietary intake of phytoestrogens and other selected nutrients, among 6,584 mothers of control infants in the National Birth Defects Prevention Study.

	Isoflavones	Lignans	Coumestrol	Total Phytoestrogens	Energy	Fiber	Carbo-hydrate	Folate
Isoflavones	1.00	0.80	0.72	0.89	0.78	0.86	0.72	0.53
Lignans	-	1.00	0.63	0.98	0.62	0.88	0.60	0.47
Coumestrol	-	-	1.00	0.71	0.62	0.62	0.62	0.48
Total Phytoestrogens	-	-	-	1.00	0.70	0.92	0.67	0.51

The food groups that were the top contributors to total phytoestrogen intake among study participants were vegetables (31%) and fruit (29%); for isoflavones, dairy (33%) and fruit (21%); for lignans, vegetables (40%) and fruit (29%); and for coumestans, fruit (55%) and dairy (18%) (Table 3). There was some variability by race-ethnicity, with the most striking difference being the greater contribution of legumes to phytoestrogen intake among Hispanics than among the other groups.

**Table 3:** Percent contribution of food groups to intake of phytoestrogens, overall and by race-ethnicity.**

	All women (n = 6,583)	White (n = 3,959)	Black (n = 739)	Hispanic (n = 1446)	Other (n = 412)
Total Phytoestrogens					
Vegetables	31	34	33	25	32
Fruit	29	26	32	31	32
Dairy	13	16	12	10	11
Legumes	10	5	3	20	10
Grains	7	7	7	7	7
Cereal	6	8	7	5	5
Meat	4	4	5	3	3
Isoflavones					
Vegetables	11	13	13	8	11
Fruit	21	17	25	25	26
Dairy	33	40	33	24	30
Legumes	14	7	4	26	14
Grains	8	9	8	7	9
Cereal	8	9	11	7	6
Meat	4	5	6	3	4
Lignans					
Vegetables	40	44	43	33	41
Fruit	29	27	32	32	32
Dairy	5	7	5	4	5
Legumes	10	5	3	19	10
Grains	7	7	7	7	6
Cereal	6	8	6	4	4
Meat	4	4	4	3	3
Coumestrol					
Vegetables	9	10	9	7	9
Fruit	55	52	61	58	60
Dairy	18	22	14	14	15
Legumes	3	1	1	5	3
Grains	7	6	6	7	7
Cereal	4	3	4	5	3
Meat	5	6	5	4	4

* Intake at least once per month during the year before pregnancy.

** Numbers may not add to 100% due to rounding.

Overall and within each racial-ethnic sub-group, oranges and broccoli were top contributors to total phytoestrogen intake; milk, oranges and cereal were top contributors to isoflavone intake; and broccoli and oranges were top contributors to lignan intake (Table 4). Refried beans also made it into these top five lists overall, which seemed to be largely driven by intake among Hispanics. For coumestans, orange juice was by far the top contributor.

**Table 4:** Food items (and their percent contribution) that were the top contributors to intake of phytoestrogens, overall and by race-ethnicity.

All women (n = 6,584)	White (n = 3,959)	Black (n = 739)	Hispanic (n = 1,446)	Other (n = 412)
Total Phytoestrogens*				
Oranges (10)	Oranges (7)*	Oranges (12)	Refried Beans (14)	Oranges (12)
Refried Beans (7)	Broccoli (7)	Yams or Sweet Potatoes (8)	Oranges (12)	Broccoli (6)
Broccoli (6)	Cereals (7)	Broccoli (6)	Avocado or Guacamole (5)	Refried Beans (6)
Cereals (6)	Skim or Low Fat Milk (7)	Cereal (6)	Broccoli (5)	Carrots, cooked (5)
Carrots, raw (5)	Carrots, Raw (6)	Whole Milk (5)	Beans or lentils, baked or dry (4)	Orange juice (4)
Isoflavones*				
Oranges (11)	Skim or low fat milk (17)	Whole milk (15)	Refried Beans (18)	Oranges (15)
Skim or low fat milk (11)	Oranges (8)	Oranges (14)	Oranges (14)	Whole milk (10)
Whole Milk (10)	Cereals (7)	Cereal (9)	Whole milk (12)	Skim or low fat milk (9)
Refried Beans (8)	Whole milk (7)	Skim or low fat milk (5)	Cereals (6)	Refried Beans (7)
Cereal (7)	Yogurt (4)	String beans (4)	Beans or lentils, baked or dried (46)	Cereal (6)
Lignans*				
Broccoli (8)	Broccoli (10)	Yams or sweet potatoes (12)	Refried Beans (14)	Oranges (10)
Oranges (8)	Carrots, raw (8)	Oranges (11)	Oranges (10)	Broccoli (9)
Carrots, raw (7)	Cereals (7)	Broccoli (9)	Broccoli (6)	Carrots, cooked (7)
Refried Beans (6)	Oranges (6)	Peaches, apricots, plums, or nectarines (7)	Avocado or guacamole (6)	Yams or sweet potatoes (6)
Carrots, cooked (6)	Other fruits, fresh, frozen, or canned (6)	Cereal (6)	Peaches, apricots, plums, or nectarines (6)	Peaches, apricots, plums, or nectarines (6)
Coumestrol*				
Orange Juice (39)	Orange juice (39)	Orange juice (43)	Orange juice (37)	Orange juice (41)
Oranges (11)	Skim or low fat milk (13)	Oranges (13)	Oranges (15)	Oranges (14)
Skim or low fat milk (8)	Oranges (8)	Whole milk (7)	Whole milk (6)	Skim or low fat milk (6)
Whole Milk (5)	Broccoli (4)	Cereal (4)	Cereal (5)	Whole milk (5)
Broccoli (4)	White bread, including pita bread (3)	Broccoli (4)	Refried Beans (4)	Broccoli (4)

* Percentage contribution of each food item to total intake of each phytoestrogen is included in parentheses; numbers may not add to 100% due to rounding

** Intake at least once per month during the year before pregnancy.

We examined the association of several maternal characteristics with total phytoestrogen intake (Table 5). Multivariable analyses indicated that women with Hispanic or 'other' race-ethnicity were much more likely to be in the highest quartile of phytoestrogen intake and less likely to be in the lowest quartile than non-Hispanic white or black women. Associations with maternal age and folic acid-containing supplements were more modest but indicated that older mothers and mothers who took supplements were more likely to be in the highest quartile than younger mothers. Maternal education was not strongly associated with phytoestrogen intake.

**Table 5:** Association of maternal characteristics with total phytoestrogen intake among 6,441 mothers of control infants in the National Birth Defects Prevention Study.

	Percent of all subjects*	No. in lowest quartile (n = 1,613)	No. in middle two quartiles (Reference, n = 3,226)	No. in highest quartile (n = 1,602)	AOR (95% CI) for lowest versus middle quartiles**	AOR (95% CI) for highest versus middle quartiles**
Maternal race-ethnicity						
White, non-Hispanic	61%	1176	2183	541	Reference	Reference
Black, non-Hispanic	11%	234	330	170	1.3 (1.1, 1.6)	1.4 (1.1, 1.8)
Hispanic	22%	140	521	756	0.3 (0.3, 0.4)	6.0 (4.9, 7.3)
Other	6%	63	192	135	0.5 (0.3, 0.7)	2.9 (2.2, 3.8)
Maternal education						
< High school	16%	188	405	469	Reference	Reference
High school	24%	449	719	409	1.1 (0.9, 1.4)	0.9 (0.7, 1.1)
> High school	59%	976	2102	724	0.7 (0.6, 0.9)	1.0 (0.8, 1.3)
Maternal age						
< 25 years	33%	587	951	592	1.7 (1.4, 2.0)	0.7 (0.6, 0.8)
25-34 years	53%	848	1758	792	Reference	Reference
35+ years	14%	178	517	218	0.5 (0.4, 0.6)	1.6 (1.3, 2.0)
Folic acid-containing supplement intake						
None	5%	66	148	93	Reference	Reference
Began in 3 mo. before pregnancy	34%	527	1221	433	0.7 (0.5, 1.1)	1.9 (1.3, 2.8)
Began in first trimester	54%	912	1647	892	0.9 (0.6, 1.3)	1.5 (1.0, 2.1)
Began after first trimester	8%	108	210	184	1.0 (0.6, 1.5)	1.4 (0.9, 2.1)

* Numbers may not add to 100% due to rounding.

**Odds ratios are adjusted for all other variables included in the table, as well as energy intake (kcals).

As noted, this study only inquired about consumption of *caffeinated *coffee and tea. During the year before pregnancy, 67% (4,421) of women drank caffeinated coffee and/or tea at least once a month (45% drank coffee, 46% drank tea). After incorporating phytoestrogens from coffee and tea into the overall phytoestrogen intake among these women, coffee was the top contributor to intake of lignans (20% of intake) and total phytoestrogens (16%) (data not shown). Tea contributed 6% of lignan intake, 7% of coumestan intake, and 4% of total phytoestrogens. The contribution of coffee and tea was otherwise negligible.

## Discussion

This study utilized published data on the phytoestrogen content of foods to estimate pre-pregnancy phytoestrogen intake among a large, population-based sample of women in the U.S. who recently delivered a baby. These estimations fill an important data gap and are particularly relevant given potential concerns about the effects of phytoestrogen intake on fetal development [12-16]. For example, genistein may affect the developing male urogenital tract, including induction of morphologic abnormalities and altered gene expression [12,29,30].

Previous studies have attempted to quantify dietary phytoestrogen intake. Estimates have tended to rely on data compiled from multiple databases, which used varied measurement techniques and measured different sets of phytoestrogens on different sets of foods. Consequently, even estimates of different phytoestrogens from the same food item may have been derived from different sources, by different methods, and many values may have remained missing. The advantage of the current study is that it could capitalize on recent values generated by a single laboratory, on a wide range of foods common to a Western diet, derived using state-of-the-art measurement techniques on several specific phytoestrogens.

Like our study, de Kleijn et al. examined phytoestrogen intake based on answers to a Willett food frequency questionnaire. Their study population included Caucasian post-menopausal women participating in the Framingham Offspring Study, and they compiled phytoestrogen values from a variety of sources [1]. In their study, the top contributors to isoflavone intake were beans and peas (26%), tea and coffee (17%), and nuts (15%), versus dairy and fruit in our study. "Soy" contributed only 3% in their study. Their top contributor to lignans was fruit (24%), versus vegetables and fruits in our study. A selected list of vegetables (broccoli, cabbage, cauliflower, lettuce and spinach) accounted for 89% of coumestan intake, versus fruit and dairy in our study. Alcohol made a minimal contribution. As the authors note, their data were most complete for fruits and vegetables, and phytoestrogen estimates were missing for many (if not all) dairy products and for several grain products. This could explain some of the differences with our results. Boker et al. examined phytoestrogen intake among Dutch women (mostly post-menopausal), using a similar approach as de Kleijn et al., but adding phytoestrogen data from some additional sources [31]. Similar to de Kleijn et al., beans and peas, tea and coffee, and nuts were important sources of isoflavone intake, but so were grain products. Grain products rather than fruits were the main contributors to lignans, and peas/beans were the main contributor to coumestrol.

We observed that total phytoestrogen intake did not differ by education but that intake in the highest quartile was more likely among women who took folic acid-containing vitamin/mineral supplements and among Hispanic women. Milder et al. observed that in the Dutch population, lignan intake was higher among people who were not overweight (BMI < 25), who had higher socioeconomic status, and who did not smoke [32]. Frankenfeld et al. observed that among post-menopausal women, plasma isoflavone levels were associated with higher intake of fruits and vegetables, lower intake of saturated fats, and higher intake of multivitamin/mineral supplements [33]. In general, these findings suggest that health behaviors and other nutritional factors, as well as race-ethnicity, may be important correlates of phytoestrogen intake that should be considered in analyses examining associations of phytoestrogen intake with health outcomes.

Some important potential limitations of assessment of phytoestrogen intake in the current study are noteworthy. The NBDPS assessment of coffee and tea intake was restricted to assessing usual intake of *caffeinated *coffee or tea *before *pregnancy, and is therefore incomplete. Among the sub-set of women who drank these beverages, coffee, and to a lesser extent tea, did make a substantial contribution to phytoestrogen intake. We also did not include phytoestrogen intake from alcoholic beverages in our estimates because information on type of wine and type of liquor, which affect phytoestrogen content, was not available. A total of 37% of mothers drank any alcoholic beverages during the month before pregnancy or the first trimester of pregnancy, but the frequency of alcohol intake tends to be relatively low, with < 3% of mothers drinking daily during the preconception time period (data not shown). Another limitation of the NBDPS food frequency questionnaire is that historically it has not included soy-based items. However, beginning with subjects who had estimated dates of delivery in 2006, the food frequency questionnaire has also included "soy milk or soy yogurt" and "tofu, tempeh or soy burgers." Preliminary data indicate that 13% of women consumed either of these products at least once per month, with only 3-4% of women consuming either food item at least once per week. This low prevalence of consumption suggests that these products are likely to be important sources of phytoestrogen intake for a relatively small proportion of the study population. Additional items that may not be consumed very frequently but are potentially important for a sub-set of individuals due to their high phytoestrogen content were also excluded from the NBDPS questionnaire; e.g., alfalfa sprouts, flax seed, and whole grain varieties of some grain products. Lack of inclusion of these items may contribute to the fact that our reported intake levels are relatively low compared to previous studies, although it should also be noted that in general there is wide variability in reported intakes [1,8,31,34]. Another potential source of measurement error is that the Kuhnle laboratory focused on food samples obtained locally; it is uncertain whether certain foods would vary between the UK and US, although Kuhnle et al. did not observe clear variability by country of origin for a select group of fruits and vegetables [35]. Given the largely descriptive nature of the current analysis, we did not explore any formal approaches to address the potential effects of measurement error. Given the retrospective study design, we could not validate our phytoestrogen estimates against a gold standard, such as serum values. However, other studies of phytoestrogen intake derived from food frequency questionnaires have demonstrated good validity when compared with serum or urine values [36,37].

## Conclusions

In this study population of women of reproductive age, the sub-group lignans and the food groups fruits and vegetables were major contributors to phytoestrogen intake, and intake varied by race-ethnicity. The advantage of the approach used for the current analysis lies in its utilization of phytoestrogen values derived from a single laboratory that used state-of-the-art measurement techniques. The database we developed can be applied directly to other studies using food frequency questionnaires, especially the Willett questionnaire. The database, combined with consistent dietary intake assessment, provides an opportunity to improve our ability to understand potential associations of phytoestrogen intake with maternal and infant health outcomes.

## Abbreviations

NBDPS: National Birth Defects Prevention Study; FFQ: food frequency questionnaire

## Competing interests

The authors declare that they have no competing interests.

## Authors' contributions

SC and MC conceived of the study; AFG and CM conducted data analyses; and all authors contributed to interpreting results and drafting the manuscript. All authors read and approved the final manuscript.
